# Nutritional Strategies to Support Performance Maintenance and Recovery in Football Under Hot Environmental Conditions: A Narrative Review

**DOI:** 10.3390/nu18111695

**Published:** 2026-05-26

**Authors:** Xincheng Dai, Shuning Liu, Dixin Zou, Songru Zou, Xiaolin Shao, Yayi Jiang, Yao Yan, Wei Jiang, Kai Zhao, Chang Liu

**Affiliations:** 1 School of Art, Beijing Sport University, Beijing 100084, China; 2023014432@bsu.edu.cn (X.D.); shaoxiaolin2022012405@bsu.edu.cn (X.S.); 2 School of Sports Science, Beijing Sport University, Beijing 100084, China; 2023013553@bsu.edu.cn; 3 State Key Laboratory for Quality Ensurance and Sustainable Use of Dao-di Herbs, Institute of Chinese Materia Medica, China Academy of Chinese Medical Sciences, Beijing 100700, China; zoudixin@163.com; 4 School of Art, Wuhan Sports University, Wuhan 430079, China; zousongru2023@163.com; 5 Graduate School of Trainers, Belarusian State University of Physical Culture, 220020 Minsk, Belarus; 13146579610@163.com; 6 China Basketball College, Beijing Sport University, Beijing 100084, China; 2024210467@bsu.edu.cn; 7 China Volleyball Academy, Beijing Sport University, Beijing 100084, China; 2430@bsu.edu.cn

**Keywords:** football, heat stress, ergogenic aids, nutritional strategies, performance maintenance, recovery, narrative review

## Abstract

Rising ambient temperatures and the increasing frequency of training and competition in hot climates have made heat stress a major challenge in football. Under such conditions, players experience greater cardiovascular and thermoregulatory strain, faster glycogen use, higher perceived exertion, and progressive impairment in repeated high-intensity actions and decision-making. These responses have intensified interest in nutritional strategies that might complement heat acclimation, hydration/electrolyte planning, cooling practices, and recovery management. This narrative review critically synthesizes current evidence on nutritional interventions that may be relevant to football performed in the heat, with emphasis on hydration and electrolyte replacement, carbohydrate–protein strategies, taurine, branched-chain amino acids (BCAAs), creatine, menthol, antioxidant- and nitrate-related approaches, and selected multi-ingredient products. Across the available literature, hydration/electrolyte planning and carbohydrate–protein feeding remain the practical foundation, menthol appears most consistently useful for perceptual cooling, creatine seems safe and potentially helpful for repeated-sprint support, and taurine is promising but still supported by relatively few trials. By contrast, evidence for BCAAs, antioxidants, nitrates, and caffeine as stand-alone heat strategies, as well as for many compound supplements, remains inconsistent, context-specific, or too indirect for strong football-specific endorsement. Overall, the evidence base remains heterogeneous in study quality, protocol design, exercise mode, and sport specificity. A substantial proportion of the available data is derived from cycling, endurance, or laboratory heat-exercise models rather than football-specific trials. Accordingly, any practical recommendation should be interpreted cautiously and embedded within broader heat-management strategies. Future work should prioritize ecologically valid randomized controlled trials in football or football-like intermittent protocols, with transparent reporting of dose, timing, perceptual outcomes, and match-relevant performance measures.

## 1. Introduction

With the progression of global climate warming, football matches held in hot climates have become increasingly frequent, and the performance and health of players in high-temperature environments have attracted widespread attention [1,2]. Hot environments exert multiple adverse effects on football players, including reductions in endurance capacity and repeated high-intensity actions, as well as impairments in cognition and decision-making [3,4]. Studies have shown that compared with temperate environments, heat significantly diminishes players’ running performance. For example, statistics from the 2022/23 UEFA Champions League season revealed that when the match temperature was ≥21 °C, both total distance covered and high-speed running distance were markedly lower than in matches played at 6–10 °C, with sprint frequency also reduced [5]. These findings indicate that hot climates make it difficult for players to maintain normal running volume and high-intensity actions.

A series of physiological responses induced by heat are the primary causes of performance decline. In hot environments, increased skin blood flow and heavy sweating facilitate heat dissipation; however, if fluid and electrolyte replacement is insufficient, dehydration and electrolyte imbalance occur, leading to reductions in plasma volume, decreased cardiac output, and elevated heart rate, thereby increasing cardiovascular strain [6]. Under controlled experimental conditions comparing exercise at 35 °C and 20 °C, athletes exhibited a more rapid rise in core body temperature and a significantly higher mean heart rate during exercise in the hot environment [2,7]. Heat also exacerbates metabolic stress, accelerates muscle glycogen breakdown, and increases reliance on anaerobic metabolism, resulting in earlier onset of fatigue [4,7]. Studies have reported that during endurance running in extreme heat approaching 40 °C, muscle glycogen depletion and blood lactate accumulation rates were markedly higher than at lower temperatures, with exercise duration significantly shortened due to metabolic disturbances [7]. Moreover, heat drives thermoregulation close to its physiological limit, and rapid elevations in core temperature can trigger protective mechanisms in the central nervous system that suppress exercise drive [3]. Subjective fatigue and thermal discomfort are also greatly increased in hot environments: during constant-intensity exercise at 35 °C, ratings of perceived exertion (RPEs) were 2–3 points higher than at 20 °C and remained consistently elevated throughout exercise [6]. Excessive body temperature and environmental heat stress further elevate the risk of exertional heat illnesses such as heat exhaustion and heat stroke [6]. Collectively, hot environments pose multiple challenges to football players, including decreased endurance, restricted high-intensity running, increased cardiovascular and thermoregulatory strain, and heightened subjective fatigue and discomfort.

To mitigate the adverse effects of heat and help maintain match performance, practitioners typically combine heat acclimation, cooling, hydration, electrolyte replacement, carbohydrate availability, and nutrition-based countermeasures [6,8,9,10,11]. Within the nutritional domain, a range of ergogenic aids have demonstrated value under thermoneutral conditions, yet their efficacy under heat stress is less certain and appears more context-dependent [12,13]. Recent syntheses suggest that caffeine and nitrate supplementation may not reliably enhance endurance performance in the heat, whereas menthol-based interventions and selected amino acid strategies may offer more promise in specific settings [13,14,15]. Importantly, such effects are intervention-specific and protocol-dependent, which limits direct extrapolation to football-specific intermittent exercise. Therefore, a careful and sport-aware synthesis of the evidence is needed before practical recommendations are extended to football performed in hot environments.

This article is intended as a narrative review informed by a structured reading of the literature rather than a formal systematic review. It focuses on football as a high-intensity intermittent sport and examines how hot environments influence performance, physiological load, and perceptual strain while critically discussing the possible role of nutritional supplementation. Emphasis is placed on taurine, branched-chain amino acids (BCAAs), creatine, menthol/menthol-containing agents, antioxidants and nitrates, carbohydrate–protein strategies, hydration/electrolyte replacement, caffeine-containing combinations, and selected multi-ingredient products. Throughout, priority is given to football-specific and exercise-in-the-heat evidence where available, while findings from related laboratory and endurance models are interpreted explicitly as indirect evidence. To improve interpretability, each strategy is discussed not only in mechanistic terms but also in relation to evidence consistency, football relevance, and practical transferability.

To improve transparency while retaining the narrative format, studies were identified through structured searches of PubMed, Web of Science, Scopus, Google Scholar, and relevant reference lists up to January 2026, followed by targeted updates up to May 2026. Search terms combined football or soccer with heat, hot environment, thermoregulation, hydration, electrolyte, carbohydrate, protein, taurine, BCAA, creatine, menthol, nitrate, antioxidant, caffeine, recovery, and multi-ingredient supplement. Priority was given to football-specific and football-like intermittent protocols; when such evidence was unavailable, studies from cycling, running, endurance, or laboratory heat-exercise models were used only as indirect evidence.

The interventions were selected because they are commonly discussed in applied sports nutrition; have plausible mechanisms related to heat strain, performance maintenance, perception, or recovery; and have at least some evidence from the exercise-in-the-heat or relevant sports-nutrition literature.

## 2. Decline in Performance and Increased Physiological Load of Football Players in Hot Environments

The direct impact of high temperatures on football players is a marked decline in performance and an increase in physiological burden [2,5]. Statistical evidence indicates that when match temperatures exceed 21–29 °C, players’ total running distance, high-speed running distance, and number of sprints during games are significantly reduced [5]. In the aforementioned study of UEFA Champions League matches, total distance covered at ≥21 °C was reduced by approximately 5–7% compared with matches at 6–10 °C, while high-speed and high-intensity running distances were also significantly lower in hotter conditions [5]. This suggests that hot climates hinder players’ ability to sustain their usual running volume and high-intensity actions. These impairments are closely associated with physiological dysfunctions under heat stress: elevated skin blood flow and excessive sweating in hot environments, if not accompanied by timely replacement of fluids and electrolytes, lead to dehydration and electrolyte imbalance, thereby reducing plasma volume and circulatory function, causing decreased cardiac output, elevated heart rate, and additional cardiovascular strain [6]. Research has shown that during exercise at 35 °C under high humidity, athletes’ core body temperature rose more rapidly, heart rates were significantly higher, and endurance capacity was notably lower than in temperate environments [2]. An experiment comparing female athletes performing interval running under hot (30–35 °C) versus temperate (15–20 °C) conditions revealed higher body temperatures and lactate levels along with shorter exercise duration in the hot conditions [7]. Heat also accelerates glycogen depletion and the accumulation of metabolic by-products, forcing earlier reliance on anaerobic metabolism, thereby reducing endurance and inducing premature fatigue [4,7]. For instance, Mohr et al. [4] observed in simulated match experiments that sprint frequency and running intensity declined more sharply during the latter stages of matches in hot environments, indicating that heat accelerates fatigue development. Moreover, under hot conditions, players’ core temperatures and heart rates are consistently higher, aggravating the overall physiological load [2].

In addition to impaired objective performance metrics, subjective fatigue and thermal discomfort are significantly exacerbated in hot environments. Athletes often experience earlier onset of fatigue, with ratings of perceived exertion (RPEs) and thermal discomfort scores markedly elevated [3]. Studies have shown that during cycling at the same intensity, in a hot environment, the RPE was already ~2 points higher at 35 °C than at 20 °C within just 10 min of exercise and remained 2–3 points higher throughout the session [6]. Heat causes athletes to feel stronger thermal stress and oppressive discomfort, and this deterioration in subjective perception further limits performance because the central nervous system may proactively reduce muscle recruitment in response to elevated core temperatures and fatigue signals, a phenomenon known as “protective fatigue” [3]. The negative effects of heat on cognition and decision-making should not be overlooked either: reports indicate that for each 1 °C increase in ambient temperature, players’ decision-making speed and accuracy decline [4]. In actual matches, the quality of technical action selection and execution may also deteriorate under hot conditions. These findings collectively suggest that heat imposes both physiological and psychological disadvantages on football players.

**Figure 1:**
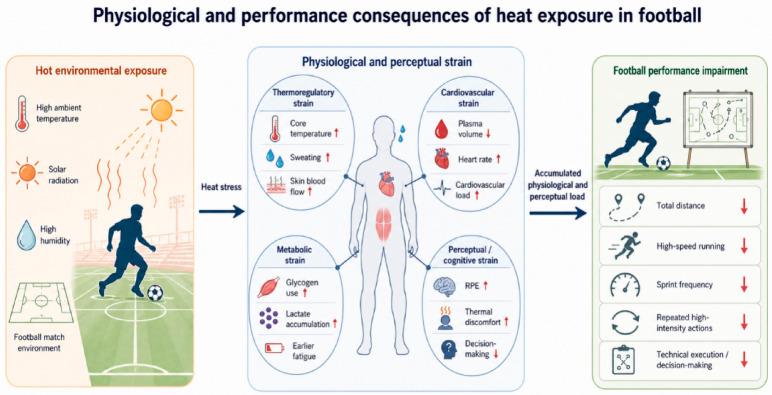
Physiological and performance consequences of heat exposure in football. The figure summarizes how hot environmental conditions increase thermoregulatory and cardiovascular strain, fluid and electrolyte losses, metabolic disturbance, perceived exertion, and thermal discomfort. These responses may contribute to earlier fatigue, reduced total and high-speed running, impaired repeated high-intensity actions, and poorer technical or cognitive performance. The framework integrates football-specific observations with broader heat-exercise physiology, exertional heat-illness guidance, heat-acclimation recommendations, and fluid-replacement principles [2,4,5,6,7,8,9]. Arrows indicate the proposed progression from environmental heat exposure to physiological and perceptual strain and then to football performance impairment; upward and downward arrows inside the figure indicate increases or decreases in the listed responses.

In summary, hot environments present football players with multiple challenges, including significantly reduced endurance, restricted high-intensity running, increased cardiovascular and thermoregulatory strain, and heightened fatigue and thermal discomfort [5,6]. Therefore, maintaining performance under heat requires effective and integrated countermeasures. In addition to systematic heat acclimation training and mid-match cooling or hydration breaks, nutritional supplementation—an accessible and practical strategy for performance regulation—has gained increasing attention. However, nutrition should be viewed as a layered system: hydration and electrolyte planning protect the physiological foundation for heat tolerance, carbohydrate availability supports intermittent high-intensity work, selected perceptual aids may reduce thermal discomfort, and recovery-oriented strategies may help players tolerate repeated heat exposure. The following sections will elaborate on the effects and mechanisms of various nutritional supplements on physical performance and the subjective perceptions of football players under hot conditions. These heat-related physiological and performance consequences are summarized in Figure 1.

## 3. Mechanisms and Intervention Effects of Nutritional Supplements Under Heat Stress

### 3.1. Taurine

Taurine is a sulfur-containing amino acid involved in osmoregulation, membrane stabilization, calcium handling, and antioxidant defense. These properties have made it a plausible candidate for attenuating heat-induced physiological strain. The currently available evidence, however, remains relatively limited and has been derived mainly from cycling or laboratory exercise models rather than football-specific trials.

In a randomized crossover trial, acute taurine ingestion (50 mg/kg body mass) before cycling in the heat improved time to exhaustion and was accompanied by favorable changes in thermoregulatory responses and perceived exertion [16]. More recently, short-term oral taurine supplementation has also been reported to modify thermoregulatory responses during low-intensity exercise in hot conditions of increasing humidity [17]. These findings are encouraging, but they should still be interpreted as preliminary because sample sizes remain small and outcome measures are heterogeneous.

Overall, taurine appears promising—particularly for perceptual and thermoregulatory outcomes—but it cannot yet be considered a football-specific standard intervention. Its role is better viewed as supportive and context-dependent until replicated in intermittent-sprint and match-like protocols.

### 3.2. Branched-Chain Amino Acids (BCAAs)

BCAAs (leucine, isoleucine, and valine) are widely used in sports nutrition because of their theorized capacity to attenuate central fatigue. Mechanistically, they may compete with tryptophan for transport across the blood–brain barrier, thereby influencing serotonin-related fatigue pathways [18]. Under heat stress, this hypothesis is attractive, but performance findings remain inconsistent.

Early work suggested that BCAA ingestion could prolong exercise capacity during heat stress [19], whereas later studies reported little or no clear benefit for prolonged exercise capacity in warm conditions [20]. More recent quantitative syntheses likewise indicate that the overall signal remains unstable, with any possible benefit likely to depend on exercise duration, feeding protocol, and outcome selection rather than representing a robust universal effect [13,15].

For football, the practical implication is therefore modest. BCAAs may still be used within broader nutrition plans, especially where athletes already tolerate them well, but the current literature does not justify treating BCAAs as a core football-in-the-heat strategy for preserving repeated high-intensity performance. The central-fatigue hypothesis is therefore mechanistically attractive, but it remains insufficiently validated for the stop–start, skill-dependent, and tactically paced demands of football in the heat.

### 3.3. Creatine

Creatine remains one of the most established ergogenic aids for repeated high-intensity exercise because it increases phosphocreatine availability and supports rapid ATP resynthesis. In hot environments, discussion has long focused on whether creatine-related body-mass gain or intracellular water retention might impair heat dissipation. Importantly, systematic review evidence does not support the claim that creatine supplementation worsens heat tolerance or hydration status [21].

Experimental studies further suggest that creatine can be used safely in the heat and may even support thermoregulatory stability when paired with adequate fluid intake [22,23]. In trained individuals, creatine loading has been associated with favorable thermoregulatory and cardiovascular responses during exercise in the heat, alongside possible benefits for repeated sprinting and high-intensity work capacity [24,25]. These effects are especially relevant to football because the sport depends heavily on short, repeated bouts of explosive effort.

Even so, creatine should not be presented as a direct anti-heat supplement. Its most defensible role is as a general performance-support aid that remains usable during hot periods of training and competition, provided that players tolerate it well and maintain sound hydration practices.

### 3.4. Menthol

Menthol is best understood as a perceptual or sensory aid rather than a metabolic supplement. By activating cold-sensitive receptors such as TRPM8, it can create a cooling sensation that improves thermal comfort and, in some settings, reduces perceived exertion. This mechanistic rationale is supported by the meta-analytic literature, which shows that menthol can enhance thermal sensation and may improve exercise performance under hot conditions, especially in endurance-type tasks [26,27].

The strongest and most consistent contribution of menthol appears to be perceptual rather than uniformly ergogenic. Individual studies have shown improved running or cycling performance with menthol mouth rinsing, beverages, or gels in the heat [28,29,30], but the magnitude of objective performance benefit varies with dose, delivery mode, exercise type, and athlete familiarity. For football, menthol is appealing because it can be integrated into halftime or cooling-break routines, yet its use should still be trialed in training rather than introduced for the first time during competition.

### 3.5. Antioxidants and Nitrates

Antioxidant- and nitrate-based supplements are often discussed in heat-stress research because hot-environment exercise elevates oxidative strain and imposes substantial cardiovascular demands. In principle, antioxidants might help limit oxidative disturbance, while nitrate-rich products may enhance nitric oxide availability and vascular function. However, the current literature does not support strong or uniform recommendations for either category in the heat [11,13].

For antioxidants, the main concern is not only uncertain acute performance benefits but also the possibility that habitual high-dose isolated supplementation may interfere with desirable redox-sensitive training adaptations. For nitrates, direct trials in temperate and hot, humid conditions have reported no clear improvement in intermittent high-intensity performance [31], and pooled evidence remains equivocal [13,15]. Accordingly, antioxidant and nitrate strategies remain biologically plausible but practically unresolved, and they should be framed as selective or experimental rather than routine football recommendations.

Recovery-oriented antioxidant evidence should also be separated from acute heat-performance evidence. For example, a recent meta-analysis on astaxanthin supplementation summarized exercise-performance and recovery biomarker outcomes, but these findings should be interpreted as general recovery evidence rather than direct support for routine football-in-the-heat supplementation [32]. In applied practice, antioxidant-rich foods or dietary patterns are more defensible than indiscriminate high-dose supplement loading, especially during periods in which training adaptation is a priority.

### 3.6. Hydration and Electrolytes

Hydration and electrolyte replacement are not optional add-ons in hot-weather football; they are the physiological foundation on which other nutritional strategies depend. Sweat losses can reduce body water, sodium availability, plasma volume, and circulatory stability, thereby increasing heart rate, perceived exertion, thermal discomfort, and the risk of exertional heat illness [6,8,9]. Because football provides only intermittent opportunities for drinking, practical plans should be prepared before match day rather than improvised during competition.

Applied hydration strategies should be individualized according to environmental conditions, the expected playing time, sweat rates, body-mass changes, gastrointestinal tolerance, and access to breaks or halftime routines [8,9,10,11]. Fluid and electrolyte planning should also be integrated with carbohydrate availability, cooling opportunities, and post-match rehydration rather than treated as a separate supplement decision.

### 3.7. Carbohydrates + Protein

Carbohydrates and protein remain the most established nutritional substrates for supporting exercise capacity, glycogen restoration, and post-exercise recovery. In hot environments, maintaining carbohydrate availability is especially important because heat can accelerate fatigue development and increase the importance of preserving pacing and repeated-effort capacity [10]. Protein does not directly attenuate heat strain, but it becomes relevant when hot-weather training or match congestion creates repeated recovery demands.

Among the strategies reviewed here, carbohydrate–protein co-ingestion has one of the strongest practical rationales. Competitive evidence shows that combined carbohydrate–protein supplementation can improve endurance-type performance in the heat [33], while the broader sports nutrition literature strongly supports its relevance for post-exercise refueling and muscle recovery. For football, the translational value is most convincing in the domains of pre-match carbohydrate availability, halftime or between-bout support where feasible, and post-match recovery rather than as a stand-alone acute ergogenic solution.

As a result, carbohydrate–protein strategies deserve a central place in applied practice. Unlike several more speculative supplements, they fit naturally within existing hydration, electrolyte, and recovery frameworks and require a less inferential leap when translated to football settings.

### 3.8. Multi-Ingredient Supplements

Multi-ingredient products are increasingly marketed for training and competition in challenging environments because they combine several putative mechanisms, such as central stimulation, substrate provision, electrolyte support, and perceptual relief. Conceptually, such combinations are attractive for football, which imposes concurrent demands on endurance, repeated sprinting, cognition, and recovery.

The evidence base, however, is heterogeneous and often highly product-specific. Some co-ingestion models, such as caffeine plus ginseng or caffeine plus taurine, have reported favorable endurance outcomes in hot or hot–humid conditions [34,35]. At the same time, pooled analyses suggest that not all multi-ingredient approaches are equally effective, and commercial blends vary substantially in dose transparency, ingredient quality, and tolerability [15].

**Figure 2:**
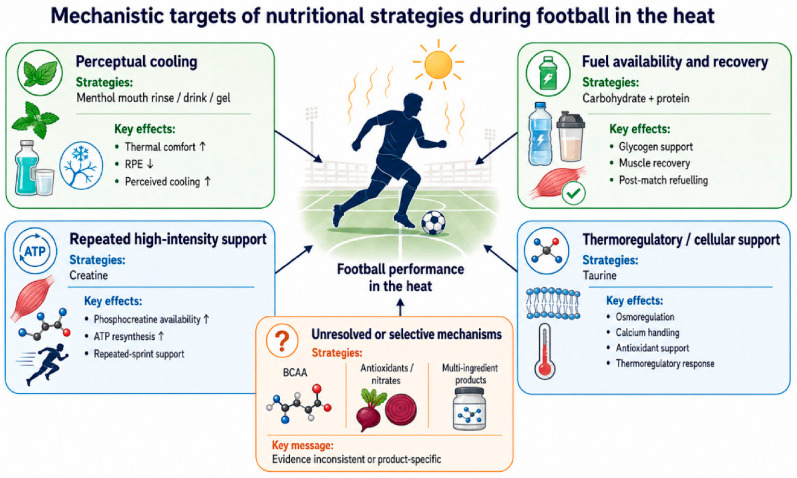
Evidence consistency and football-specific transferability of nutritional strategies for exercise in the heat. Nutritional strategies are positioned qualitatively according to two dimensions: consistency of evidence in hot-environment exercise and practical transferability to football-specific intermittent high-intensity demands. The appraisal is narrative rather than a formal GRADE certainty rating, and it reflects both direct evidence from heat-exercise studies and indirect evidence from the broader sports nutrition, supplement, and thermoregulation literature [12,13,14,15,16,17,21,22,23,24,25,26,27,28,29,30,31,32,33,34,35,36,37,38]. Arrows pointing toward the central player indicate the proposed contribution of each nutritional strategy to performance support under heat stress; upward arrows inside boxes indicate increases or improvements in the listed mechanisms or perceptual outcomes.

Therefore, multi-ingredient supplements should not be treated as inherently superior to well-implemented basic strategies. Their use is most defensible when the formulation is transparent, the player has already tolerated it in training, and the ingredients included are individually justified rather than simply bundled for marketing appeal. Caffeine-containing products deserve particular caution because caffeine is widely used in football, but heat-focused evidence suggests inconsistent benefits and possible thermoregulatory concerns depending on dose, protocol, and environmental conditions [14]. Overall, the relative evidence consistency and football-specific transferability of the strategies discussed above are summarized in Figure 2, and their applied interpretation is further compared in Table 1.

## 4. Practical Considerations for Applied Use in Football

**Figure 3:**
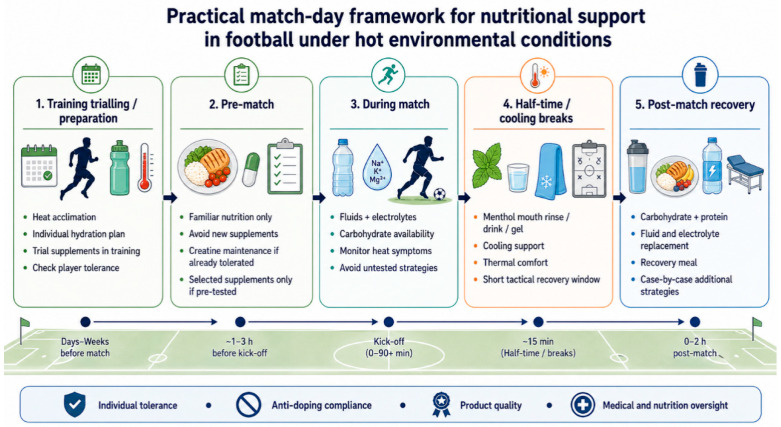
Practical match-day framework for nutritional support in football under hot environmental conditions. The framework organizes applied decision-making across training trialing/preparation; pre-match, during-match, halftime or cooling breaks; and post-match recovery. It emphasizes that nutritional strategies should be pre-tested, individualized, and embedded within heat acclimation, hydration planning, cooling logistics, carbohydrate availability, recovery nutrition, and product-quality or anti-doping checks [6,8,9,10,11,30,33,39,40].

Based on the current literature, the following points should be interpreted as cautious practical considerations rather than prescriptive supplementation protocols for football players competing in hot environments. These considerations are organized into a practical match-day framework in Figure 3.

For applied use, the evidence is most clearly interpreted as a foundation-first hierarchy. Hydration/electrolyte planning and carbohydrate–protein recovery sit at the base; menthol may be considered a perceptual adjunct during cooling opportunities; creatine and taurine remain plausible but less football-specific; and BCAAs, antioxidants, nitrates, caffeine-based combinations, and multi-ingredient products should be used selectively rather than routinely.

(1) Build the foundation: prioritize heat acclimation, individualized hydration/electrolyte planning, carbohydrate availability, and basic recovery nutrition. Nutritional supplements are not a substitute for core heat-management practices. Teams should first establish appropriate heat-acclimation procedures, individualized fluid plans, and monitoring of body-mass loss before relying on supplements to support performance in the heat [6,8,9,10,11].

(2) Consider supplement combinations conservatively: multi-ingredient strategies may be promising, but the current evidence base is still limited and not uniformly football-specific [15]. Accordingly, combinations should be selected cautiously, with preference given to ingredients that are mechanistically justified and already tolerated well by the player in training.

Before competition, low-to-moderate doses of selected supplements—such as caffeine, taurine, or other familiar ingredients—may be considered in carefully monitored settings. However, the football-specific evidence base under heat remains limited, and fixed pre-match formulas should not be generalized across players.

Creatine may be continued as part of an established loading or maintenance routine in players who have already tolerated it well, but acute pre-match loading is not advisable. Its main practical value is more likely to relate to repeated-sprint support and training continuity than to direct acute heat protection.

(3) During the match: hydration and electrolyte replacement should remain the priority. Menthol-containing drinks, gels, or mouth-rinse strategies may be considered during halftime or cooling breaks to improve thermal comfort and perceived exertion, but their use should be pre-tested in training and kept within well-tolerated concentrations [27,28].

(4) Post-match recovery: early carbohydrate–protein feeding, together with fluid and electrolyte replacement, currently has the strongest practical rationale for recovery after hot-environment exercise [10,33,40]. Additional agents such as taurine, antioxidant-rich foods, or recovery-oriented supplements may be considered on a case-by-case basis, but routine post-match use cannot yet be recommended as a football-specific standard.

Recent targeted updates further support this cautious, football-specific interpretation. Recent caffeine–taurine co-ingestion data in the heat reported no improvement in time to exhaustion despite greater ventilatory and metabolic demand, reinforcing the cautious interpretation of caffeine-containing multi-ingredient products [41]. Tournament-specific guidance for the 2026 Men’s FIFA World Cup similarly emphasizes evidence-based heat-mitigation strategies while acknowledging that direct football-specific data remain limited [42]. Match-based data from highly trained male youth football players competing in 26–42 °C conditions showed high peak core temperatures (mean 39.2 °C; range 37.9–40.1 °C), with heart rate, sweat loss, body-mass loss, and match running associated with greater heat strain [43].

(5) Individualization and monitoring remain essential. Responses to supplements vary considerably among players, so dosing and timing should be refined through repeated use in training, accompanied by monitoring of subjective responses (e.g., RPE and thermal comfort) and practical physiological indicators (e.g., body-mass change, fluid intake, and heart rate). New supplement strategies should not be introduced for the first time in competition.

(6) Anti-doping compliance and product quality must be ensured. Although commonly used supplements such as creatine, BCAAs, taurine, and caffeine are not prohibited by WADA, excessive intake, contaminated products, and poorly regulated herbal preparations still pose practical risks. Teams should therefore use reputable manufacturers and integrate supplementation decisions with medical and nutritional oversight. Supplement decisions should also follow high-performance sport consensus guidance that emphasizes evidence of benefits, product quality, contamination risk, and individual athlete needs [39].

(7) Nutritional strategies should be integrated with tactical and logistical support. Match pacing, substitutions, cooling breaks, sideline cooling resources, and environmental scheduling remain central to performance protection in the heat. Supplementation is best viewed as one component within this broader heat-management framework rather than as a stand-alone solution.

## 5. Conclusions

Hot environments impose substantial challenges on football performance by increasing physiological strain, worsening thermal perception, and reducing the capacity to sustain repeated high-intensity actions [5,6]. The literature reviewed here suggests that some nutritional strategies—particularly menthol for perceptual relief, carbohydrate–protein intake for fueling and recovery, creatine for repeated-sprint support, and taurine for selected thermoregulatory outcomes—may offer practical value. Nevertheless, the strength of evidence differs markedly across supplements, and a considerable proportion of the available data still comes from general exercise-in-the-heat models rather than football-specific trials.

Accordingly, current recommendations should remain restrained. Hydration/electrolyte planning and carbohydrate–protein strategies should remain the most immediately transferable foundation; menthol appears to have useful adjunctive value; and taurine and creatine are promising but still require more football-specific confirmation. Evidence for BCAAs, antioxidants, nitrates, caffeine as a stand-alone heat strategy, and many multi-ingredient products is less stable and should be interpreted cautiously. Future studies should prioritize ecologically valid football protocols, transparent intervention reporting, and rigorous randomized controlled designs to clarify which strategies genuinely improve match-relevant performance and recovery in the heat.

In practical terms, nutritional supplementation should be positioned as an adjunct torather than a replacement for, heat acclimation, individualized hydration planning, cooling strategies, and match-management decisions. For coaches and sports science staff, the priority should be to secure the fundamentals of fluid–electrolyte balance, carbohydrate availability, and recovery nutrition before introducing adjunctive perceptual or ergogenic aids. Any supplement strategy should be trialed during training, tailored to individual tolerance and match logistics, and screened for anti-doping and product-quality risks. Until stronger football-specific evidence becomes available, a restrained, foundation-first approach remains the most defensible way to apply nutritional support in hot environmental conditions.

## Figures and Tables

**Table 1:** Comparative appraisal of nutritional strategies discussed in this review.

Strategy	Primary Heat-Related Rationale	Current Evidence Profile	Football-Specific Transferability	Applied Interpretation
Hydration and electrolytes	Supports fluid balance, sodium replacement, plasma volume maintenance, thermoregulation, and heat-illness risk reduction.	Foundational heat-management strategy supported by consensus guidance; football-specific implementation depends on sweat rates, access to breaks, and the environmental context.	High. Directly relevant to football training and match play in the heat, although exact intake plans must be individualized.	Should be planned before match day and integrated with carbohydrate intake, cooling opportunities, and post-match rehydration.
Carbohydrate + protein	Supports carbohydrate availability, glycogen restoration, muscle repair, and post-exercise recovery.	Strongest applied rationale among the strategies reviewed; direct heat-exercise evidence is supported by the broader sports nutrition literature.	Moderate to high. Highly relevant to match fueling, between-bout support, and post-match recovery.	Should remain a central practical strategy, especially for post-match refueling and recovery in hot conditions.
Menthol	Provides perceptual cooling through cold-sensitive sensory pathways, potentially reducing thermal discomfort and perceived exertion.	Relatively consistent benefits for thermal sensation and perceptual outcomes; objective performance effects are more context-dependent.	Moderate. Particularly relevant to halftime, cooling breaks, and short windows for perceptual relief.	Useful as an adjunctive cooling-support strategy but should be trialed in training before competition.
Creatine	Supports phosphocreatine availability and rapid ATP resynthesis during repeated high-intensity efforts.	Safety data in the heat are generally reassuring; performance evidence is mainly indirect for football-specific heat exposure.	Moderate. Relevant to repeated-sprint and high-intensity demands but not a direct anti-heat intervention.	Reasonable to continue in players who already tolerate it; avoid acute loading immediately before hot-weather competition.
Taurine	May support osmoregulation, membrane stability, calcium handling, antioxidant defense, and thermoregulatory responses.	Promising but limited; most evidence comes from small cycling or laboratory heat-exercise studies.	Low to moderate. Mechanistically plausible but not yet validated in football-specific intermittent protocols.	May be considered on an individual basis after training trialing; not yet a standard football-in-the-heat recommendation.
BCAAs	Proposed to attenuate central fatigue through competition with tryptophan transport and serotonin-related pathways.	Inconsistent; early positive findings have not been reproduced consistently across heat-exercise studies.	Low. Limited relevance to repeated-sprint or match-specific football outcomes.	Optional only within broader nutrition routines; should not be promoted as a core heat-performance strategy.
Antioxidants/nitrates	Potential oxidative-stress modulation and vascular or nitric-oxide-related support.	Heterogeneous and unresolved; nitrate evidence for intermittent high-intensity performance in heat is not convincing.	Low. Biological rationale exists, but football-specific practical transfer remains weak.	Use selectively and cautiously; routine supplementation for football in the heat is not currently supported.
Multi-ingredient products	Attempts to combine several mechanisms, including substrate provision, stimulation, electrolyte support, perceptual relief, and recovery.	Highly formulation-specific; evidence is difficult to generalize because products vary in dose transparency, ingredient quality, and tolerability.	Low to moderate. Potentially relevant but strongly dependent on the exact formulation and player tolerance.	Consider only when the formulation is transparent, individually justified, anti-doping compliant, and already tolerated in training.

Note: The appraisal is based on a narrative synthesis of the evidence discussed in Section 3.1, Section 3.2, Section 3.3, Section 3.4, Section 3.5, Section 3.6, Section 3.7 and Section 3.8 and is intended to guide applied interpretation rather than provide a formal certainty ranking. The “current evidence profile” reflects the consistency and directness of evidence from hot-environment exercise studies and the relevant sports nutrition literature, whereas “football-specific transferability” reflects relevance to intermittent high-intensity football demands, match-day feasibility, and practical implementation. BCAAs, branched-chain amino acids; EHS, exertional heat stress; ATP, adenosine triphosphate; RPE, rating of perceived exertion; TRPM8, transient receptor potential melastatin 8. Key supporting sources include supplement consensus guidance, heat-exercise reviews and meta-analyses, and selected intervention trials [10,12,13,14,15,16,17,18,19,20,21,22,23,24,25,26,27,28,29,30,31,32,33,34,35,36,37,38,39,40].

## Data Availability

No new datasets or materials were generated or analyzed in this narrative review.
